# 2-Bromo-*p*-terphen­yl

**DOI:** 10.1107/S1600536810007348

**Published:** 2010-03-03

**Authors:** Suk-Hee Moon, Heesook Yoon, Youngjin Kang

**Affiliations:** a Subdivision of Food Science, Kyungnam College of Information and Technology, Busan 616-701, Republic of Korea; b Division of Science Education, Kangwon National University, Chuncheon 200-701, Republic of Korea

## Abstract

In the title compound, C_18_H_13_Br, the dihedral angles between the mean planes of the central benzene ring and the mean planes of the outer phenyl and bromo­phenyl rings are 33.47 (8) and 66.35 (8)°, respectively. In the crystal, weak C—H⋯π and inter­molecular Br⋯Br [3.5503 (15) Å] inter­actions contribute to the stabilization of the packing.

## Related literature

For the synthesis, see: France *et al.* (1938 ▶); Tadashi *et al.* (1962 ▶). For the Suzuki coupling reaction, see: Miyaura & Suzuki (1995 ▶). For cross-coupling reactions of *o*-halogenated arenes, see: Ishikawa & Manabe (2007 ▶). For organic light-emitting diodes, see: Kim *et al.* (2008 ▶). For related structures, see: Jones *et al.* (2005 ▶); Liang (2008 ▶); MacNeil & Decken (1999 ▶); Politzer *et al.* (2007 ▶).
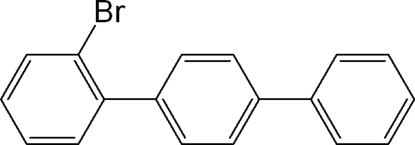


## Experimental

### 

#### Crystal data

C_18_H_13_Br
*M* *_r_* = 309.19
Monoclinic, 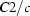

*a* = 27.039 (10) Å
*b* = 7.597 (3) Å
*c* = 18.907 (7) Å
β = 133.650 (5)°
*V* = 2810 (2) Å^3^
*Z* = 8
Mo *K*α radiation
μ = 2.91 mm^−1^
*T* = 293 K
0.30 × 0.25 × 0.20 mm

#### Data collection

Bruker SMART CCD area-detector diffractometer
Absorption correction: multi-scan (*SADABS*; Sheldrick, 1999 ▶) *T*_min_ = 0.476, *T*_max_ = 0.594
13933 measured reflections
3503 independent reflections
2246 reflections with *I* > 2σ(*I*)
*R*_int_ = 0.028

#### Refinement

*R*[*F* ^2^ > 2σ(*F* ^2^)] = 0.034
*wR*(*F* ^2^) = 0.092
*S* = 1.02
3503 reflections
172 parameters
H-atom parameters constrained
Δρ_max_ = 0.43 e Å^−3^
Δρ_min_ = −0.65 e Å^−3^

### 

Data collection: *SMART* (Bruker, 2000 ▶); cell refinement: *SAINT-Plus* (Bruker, 2000 ▶); data reduction: *SAINT-Plus*; program(s) used to solve structure: *SHELXS97* (Sheldrick, 2008 ▶); program(s) used to refine structure: *SHELXL97* (Sheldrick, 2008 ▶); molecular graphics: *SHELXTL* (Sheldrick, 2008 ▶); software used to prepare material for publication: *SHELXTL*.

## Supplementary Material

Crystal structure: contains datablocks I, global. DOI: 10.1107/S1600536810007348/jj2023sup1.cif

Structure factors: contains datablocks I. DOI: 10.1107/S1600536810007348/jj2023Isup2.hkl

Additional supplementary materials:  crystallographic information; 3D view; checkCIF report

## Figures and Tables

**Table 1:** C—H⋯π inter­actions (Å, °) *Cg*2 and *Cg*3 are the centroids of the C7–C12 and C13–C18 rings, respectively.

*D*—H⋯*A*	*D*—H	H⋯*A*	*D*⋯*A*	*D*—H⋯*A*
C2—H2⋯*Cg*3^i^	0.93	2.84	3.778 (4)	148
C14—H14⋯*Cg*2^ii^	0.93	2.97	3.658 (5)	147

Symmetry codes: (i) 
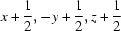
; (ii) 
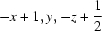
.

**Fig. 1.**
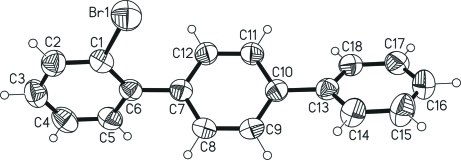
Molecular structure of the title compound, showing the atom-numbering scheme. Displacement ellipsoids are drawn at the 50% probability level.

**Fig. 2.**
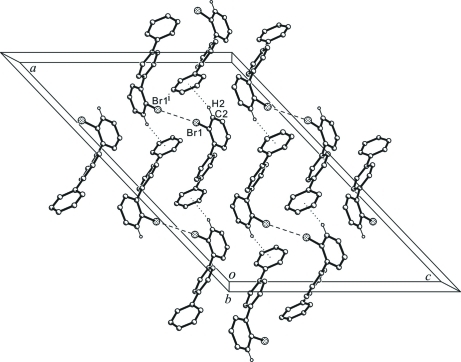
Weak Br···Br and C—H···Cg π-ring interactions (dashed lines) in the title compound.

## supplementary crystallographic information

### Comment

Palladium-catalyzed cross-coupling reactions of aryl halides with arylboronic acids, often referred as Suzuki coupling reactions, are versatile synthetic methods for the preparation of unsymmetrical biaryls. The Suzuki coupling reactions have been applied extensively in the synthesis of natural products, nucleoside analogues, and pharmaceuticals (Miyaura & Suzuki, 1995). Cross-coupling reactions of *o*-halogenated arenes are very useful synthetically, if the halogen atom is converted to other functional groups, such as carbazole, anthracene and fluorene (Ishikawa & Manabe, 2007).  Anthracene based terphenyl derivatives are widely used as emitting and/or host materials in organic light-emitting diodes (OLEDs) (Kim *et al.*, 2008).  To be good host materials in OLEDs, the host must have larger energy gap between the HOMO (Highest Occupied Molecular Orbital) and the LUMO (Lowest Unoccupied Molecular orbital) than in a dopant, because energy transfer occurs from host to dopant.

The introduction of substituents at the *ortho*-position of biary and terphenyl groups is often used in the preparation of an efficient host with a wide band gap, because the substitients suppress effective π-conjugation throughout the whole molecule.  Therefore, the structures of biaryl and terphenyl derivatives bearing a halogen atom at the *ortho* -position are attractive as good precursors to materials oriented chemists and physicists.  The title compound, C_18_H_13_Br,(I), was synthesized by the Pd-catalyzed cross coupling of 4-biphenylboronic acid with 1-bromo-2-iodobenzene in the presence of base (Na_2_CO_3_).

The dihedral angles between the mean planes of the central phenyl ring (C7-C12) and the mean planes of the outer phenyl (C13-C18) and the brominated phenyl (C1-C6) rings, are 33.47 (8)° and 66.35 (8)°, respectively (Fig. 1). All bond lengths and bond angles are normal and comparable to those observed in similar structures (MacNeil & Decken, 1999; Jones *et al.*, 2005).

Weak C–H···Cg π-ring interactions are observed [C2–H2···Cg3; H2···Cg3 =   Å; C2–H2···Cg = 148°, C2···Cg3-H2 = 3.778 (4) Å; 1/2+x,1/2-y,1/2+z and C14–H14···Cg2; H14···Cg2 = 2.97 Å; C14–H14···Cg2 = 147°, C14···Cg2–H14 = 3.658 (5) Å; 1-x,y,1/2-z; where Cg2 and Cg3 are the centroids of C7—C12 and C13—C18, respectively] (Fig. 2).  Weak Br1···Br1 interactions also exist (3.5503 (15) Å; Politzer *et al.*, 2007; Liang, 2008) and along with C–H···Cg π-ring interactions contribute to the stabilization of crystal packing.

### Experimental

A mixture of 4-biphenyboronicacid (0.1 mol), 1-bromo-2iodobenzene (0.1 mol), Na_2_CO_3_ (0.6 mol, 2M in H_2_O), and Pd(PPh_3_)_4_ (5% mol) was refluxed for 12 h.  After being cooled to room temperature, the reaction mixture was quenched by water. The aqueous layer was extracted with CH_2_Cl_2_, and the combined organic layers were sequentially washed with saturated aqueous NaCl (20 mL), dried with MgSO_4_, filtered, and concentrated under reduced pressure.  The residue was purified by silica gel column chromatography using CH_2_Cl_2_ and  hexane to give the titled compound as a colorless solid. Analytical data (France, *et al.*, 1938; Tadashi, *et al.*, 1962). ^1^H NMR (CDCl_3_, 300 MHz): 7.65 (m, 5H), 7.51 (m, 4H), 7.40 (m, 3H), 7.21 (m, 1H); MS(EI, *m/z*): 309 [M^+^]. Slow evaporation of CH_2_Cl_2_ gave suitable single crystals for X-ray analysis.

### Refinement

All H-atoms were positioned geometrically and refined using a riding model with C–H = 0.93 Å, and with *U*_iso_(H) = 1.2*U*_eq_(C).

### Crystal data

**Table:**

C_18_H_13_Br	*F*(000) = 1248
*M**_r_* = 309.19	*D*_x_ = 1.462 Mg m^−^^3^
Monoclinic, *C*2/*c*	Mo *K*α radiation, λ = 0.71073 Å
Hall symbol: -C 2yc	Cell parameters from 3654 reflections
*a* = 27.039 (10) Å	θ = 2.1–28.4°
*b* = 7.597 (3) Å	µ = 2.91 mm^−^^1^
*c* = 18.907 (7) Å	*T* = 293 K
β = 133.650 (5)°	Block, colorless
*V* = 2810 (2) Å^3^	0.30 × 0.25 × 0.20 mm
*Z* = 8	

### Data collection

**Table:**

Bruker SMART CCD area-detector diffractometer	3503 independent reflections
Radiation source: fine-focus sealed tube	2246 reflections with *I* > 2σ(*I*)
graphite	*R*_int_ = 0.028
φ and ω scans	θ_max_ = 28.4°, θ_min_ = 2.1°
Absorption correction: multi-scan (*SADABS*; Sheldrick, 1999)	*h* = −34→36
*T*_min_ = 0.476, *T*_max_ = 0.594	*k* = −9→10
13933 measured reflections	*l* = −25→25

### Refinement

**Table:**

Refinement on *F*^2^	Primary atom site location: structure-invariant direct methods
Least-squares matrix: full	Secondary atom site location: difference Fourier map
*R*[*F*^2^ > 2σ(*F*^2^)] = 0.034	Hydrogen site location: inferred from neighbouring sites
*wR*(*F*^2^) = 0.092	H-atom parameters constrained
*S* = 1.02	*w* = 1/[σ^2^(*F*_o_^2^) + (0.0341*P*)^2^ + 2.6031*P*] where *P* = (*F*_o_^2^ + 2*F*_c_^2^)/3
3503 reflections	(Δ/σ)_max_ = 0.001
172 parameters	Δρ_max_ = 0.43 e Å^−^^3^
0 restraints	Δρ_min_ = −0.65 e Å^−^^3^

### Special details

**Table:**

Geometry. All esds (except the esd in the dihedral angle between two l.s. planes) are estimated using the full covariance matrix. The cell esds are taken into account individually in the estimation of esds in distances, angles and torsion angles; correlations between esds in cell parameters are only used when they are defined by crystal symmetry. An approximate (isotropic) treatment of cell esds is used for estimating esds involving l.s. planes.
Refinement. Refinement of *F*^2^ against ALL reflections. The weighted *R*-factor wR and goodness of fit *S* are based on *F*^2^, conventional *R*-factors *R* are based on *F*, with *F* set to zero for negative *F*^2^. The threshold expression of *F*^2^ > σ(*F*^2^) is used only for calculating *R*-factors(gt) etc. and is not relevant to the choice of reflections for refinement. *R*-factors based on *F*^2^ are statistically about twice as large as those based on *F*, and *R*- factors based on ALL data will be even larger.

### Fractional atomic coordinates and isotropic or equivalent isotropic displacement parameters (Å^2^)

**Table:**

	*x*	*y*	*z*	*U*_iso_*/*U*_eq_	
Br1	0.721104 (15)	0.25216 (4)	0.56047 (2)	0.07170 (13)	
C1	0.69010 (12)	0.4341 (3)	0.59063 (16)	0.0458 (5)	
C2	0.73645 (13)	0.5631 (3)	0.65377 (18)	0.0565 (6)	
H2	0.7807	0.5590	0.6797	0.068*	
C3	0.71685 (15)	0.6982 (3)	0.67834 (19)	0.0613 (7)	
H3	0.7478	0.7861	0.7210	0.074*	
C4	0.65143 (16)	0.7030 (3)	0.6398 (2)	0.0616 (7)	
H4	0.6384	0.7930	0.6575	0.074*	
C5	0.60498 (13)	0.5756 (3)	0.57514 (18)	0.0552 (6)	
H5	0.5606	0.5819	0.5489	0.066*	
C6	0.62281 (11)	0.4369 (3)	0.54788 (15)	0.0434 (5)	
C7	0.57071 (12)	0.3039 (3)	0.47552 (17)	0.0446 (5)	
C8	0.51546 (12)	0.3516 (3)	0.37957 (17)	0.0519 (6)	
H8	0.5104	0.4684	0.3609	0.062*	
C9	0.46779 (12)	0.2289 (3)	0.31111 (18)	0.0522 (6)	
H9	0.4310	0.2648	0.2472	0.063*	
C10	0.47356 (11)	0.0529 (3)	0.33573 (16)	0.0441 (5)	
C11	0.52859 (12)	0.0067 (3)	0.43275 (16)	0.0520 (6)	
H11	0.5334	−0.1097	0.4519	0.062*	
C12	0.57609 (13)	0.1291 (3)	0.50101 (17)	0.0524 (6)	
H12	0.6124	0.0940	0.5653	0.063*	
C13	0.42539 (11)	−0.0832 (3)	0.26121 (16)	0.0453 (5)	
C14	0.39663 (13)	−0.0664 (4)	0.16585 (18)	0.0592 (6)	
H14	0.4062	0.0328	0.1483	0.071*	
C15	0.35413 (16)	−0.1948 (4)	0.0972 (2)	0.0730 (8)	
H15	0.3359	−0.1824	0.0340	0.088*	
C16	0.33855 (14)	−0.3410 (4)	0.1214 (2)	0.0703 (8)	
H16	0.3098	−0.4273	0.0749	0.084*	
C17	0.36580 (13)	−0.3588 (3)	0.2151 (2)	0.0598 (7)	
H17	0.3550	−0.4569	0.2316	0.072*	
C18	0.40904 (13)	−0.2318 (3)	0.28455 (19)	0.0519 (6)	
H18	0.4275	−0.2457	0.3477	0.062*	

### Atomic displacement parameters (Å^2^)

**Table:**

	*U*^11^	*U*^22^	*U*^33^	*U*^12^	*U*^13^	*U*^23^
Br1	0.0723 (2)	0.0729 (2)	0.0885 (2)	0.00301 (14)	0.0625 (2)	−0.01335 (15)
C1	0.0545 (14)	0.0446 (12)	0.0456 (13)	0.0024 (11)	0.0373 (12)	0.0014 (10)
C2	0.0533 (14)	0.0587 (15)	0.0542 (14)	−0.0045 (12)	0.0358 (13)	0.0008 (12)
C3	0.0735 (19)	0.0519 (14)	0.0538 (16)	−0.0128 (13)	0.0421 (15)	−0.0089 (12)
C4	0.085 (2)	0.0461 (14)	0.0686 (17)	0.0016 (13)	0.0585 (17)	−0.0044 (12)
C5	0.0604 (15)	0.0474 (14)	0.0635 (16)	0.0056 (12)	0.0449 (14)	0.0019 (12)
C6	0.0498 (13)	0.0417 (12)	0.0408 (12)	0.0028 (10)	0.0320 (11)	0.0049 (9)
C7	0.0478 (13)	0.0444 (12)	0.0457 (13)	0.0023 (10)	0.0338 (12)	0.0007 (10)
C8	0.0521 (14)	0.0397 (13)	0.0531 (15)	0.0063 (11)	0.0322 (13)	0.0065 (11)
C9	0.0461 (13)	0.0519 (15)	0.0438 (13)	0.0079 (11)	0.0254 (11)	0.0078 (11)
C10	0.0424 (12)	0.0472 (12)	0.0451 (12)	0.0016 (10)	0.0312 (11)	0.0007 (10)
C11	0.0570 (15)	0.0439 (13)	0.0462 (14)	0.0000 (11)	0.0323 (13)	0.0063 (11)
C12	0.0528 (14)	0.0498 (14)	0.0406 (12)	−0.0006 (11)	0.0270 (12)	0.0044 (11)
C13	0.0404 (12)	0.0480 (13)	0.0467 (13)	0.0030 (10)	0.0298 (11)	0.0010 (10)
C14	0.0581 (15)	0.0671 (17)	0.0520 (15)	−0.0102 (13)	0.0378 (13)	−0.0029 (13)
C15	0.0733 (19)	0.085 (2)	0.0504 (16)	−0.0171 (16)	0.0388 (16)	−0.0123 (15)
C16	0.0628 (18)	0.0634 (18)	0.0646 (19)	−0.0129 (14)	0.0363 (16)	−0.0165 (14)
C17	0.0541 (15)	0.0479 (14)	0.0654 (17)	−0.0005 (12)	0.0367 (14)	0.0014 (13)
C18	0.0493 (13)	0.0500 (14)	0.0504 (14)	0.0041 (11)	0.0322 (12)	0.0047 (11)

### Geometric parameters (Å, °)

**Table:**

Br1—C1	1.897 (2)	C9—H9	0.9300
C1—C2	1.375 (3)	C10—C11	1.391 (3)
C1—C6	1.391 (3)	C10—C13	1.489 (3)
C2—C3	1.375 (4)	C11—C12	1.376 (3)
C2—H2	0.9300	C11—H11	0.9300
C3—C4	1.372 (4)	C12—H12	0.9300
C3—H3	0.9300	C13—C18	1.390 (3)
C4—C5	1.374 (4)	C13—C14	1.391 (3)
C4—H4	0.9300	C14—C15	1.378 (4)
C5—C6	1.397 (3)	C14—H14	0.9300
C5—H5	0.9300	C15—C16	1.374 (4)
C6—C7	1.489 (3)	C15—H15	0.9300
C7—C8	1.383 (3)	C16—C17	1.377 (4)
C7—C12	1.386 (3)	C16—H16	0.9300
C8—C9	1.379 (3)	C17—C18	1.380 (3)
C8—H8	0.9300	C17—H17	0.9300
C9—C10	1.389 (3)	C18—H18	0.9300
			
C2—C1—C6	122.4 (2)	C9—C10—C11	117.1 (2)
C2—C1—Br1	116.95 (18)	C9—C10—C13	121.9 (2)
C6—C1—Br1	120.61 (17)	C11—C10—C13	120.9 (2)
C3—C2—C1	119.5 (2)	C12—C11—C10	121.5 (2)
C3—C2—H2	120.2	C12—C11—H11	119.2
C1—C2—H2	120.2	C10—C11—H11	119.2
C4—C3—C2	119.8 (2)	C11—C12—C7	121.1 (2)
C4—C3—H3	120.1	C11—C12—H12	119.5
C2—C3—H3	120.1	C7—C12—H12	119.5
C3—C4—C5	120.4 (2)	C18—C13—C14	118.1 (2)
C3—C4—H4	119.8	C18—C13—C10	121.7 (2)
C5—C4—H4	119.8	C14—C13—C10	120.2 (2)
C4—C5—C6	121.5 (2)	C15—C14—C13	120.8 (3)
C4—C5—H5	119.2	C15—C14—H14	119.6
C6—C5—H5	119.2	C13—C14—H14	119.6
C1—C6—C5	116.3 (2)	C16—C15—C14	120.4 (3)
C1—C6—C7	123.5 (2)	C16—C15—H15	119.8
C5—C6—C7	120.2 (2)	C14—C15—H15	119.8
C8—C7—C12	117.8 (2)	C15—C16—C17	119.6 (3)
C8—C7—C6	120.5 (2)	C15—C16—H16	120.2
C12—C7—C6	121.7 (2)	C17—C16—H16	120.2
C9—C8—C7	121.2 (2)	C16—C17—C18	120.4 (3)
C9—C8—H8	119.4	C16—C17—H17	119.8
C7—C8—H8	119.4	C18—C17—H17	119.8
C8—C9—C10	121.3 (2)	C17—C18—C13	120.8 (2)
C8—C9—H9	119.3	C17—C18—H18	119.6
C10—C9—H9	119.3	C13—C18—H18	119.6
			
C6—C1—C2—C3	1.8 (4)	C8—C9—C10—C13	−175.5 (2)
Br1—C1—C2—C3	−179.94 (19)	C9—C10—C11—C12	−1.3 (3)
C1—C2—C3—C4	0.0 (4)	C13—C10—C11—C12	175.5 (2)
C2—C3—C4—C5	−1.4 (4)	C10—C11—C12—C7	0.2 (4)
C3—C4—C5—C6	1.0 (4)	C8—C7—C12—C11	1.0 (4)
C2—C1—C6—C5	−2.2 (3)	C6—C7—C12—C11	−178.2 (2)
Br1—C1—C6—C5	179.65 (16)	C9—C10—C13—C18	−149.7 (2)
C2—C1—C6—C7	176.8 (2)	C11—C10—C13—C18	33.6 (3)
Br1—C1—C6—C7	−1.4 (3)	C9—C10—C13—C14	31.9 (3)
C4—C5—C6—C1	0.8 (3)	C11—C10—C13—C14	−144.8 (2)
C4—C5—C6—C7	−178.2 (2)	C18—C13—C14—C15	−1.1 (4)
C1—C6—C7—C8	−113.1 (3)	C10—C13—C14—C15	177.4 (3)
C5—C6—C7—C8	65.9 (3)	C13—C14—C15—C16	1.0 (5)
C1—C6—C7—C12	66.1 (3)	C14—C15—C16—C17	−0.1 (5)
C5—C6—C7—C12	−115.0 (3)	C15—C16—C17—C18	−0.7 (4)
C12—C7—C8—C9	−0.9 (4)	C16—C17—C18—C13	0.6 (4)
C6—C7—C8—C9	178.2 (2)	C14—C13—C18—C17	0.3 (3)
C7—C8—C9—C10	−0.3 (4)	C10—C13—C18—C17	−178.2 (2)
C8—C9—C10—C11	1.4 (4)		

### Hydrogen-bond geometry (Å, °)

**Table:**

Cg2 and Cg3 are the centroids of the C7–C12 and C13–C18 rings, respectively.

**Table:**

*D*—H···*A*	*D*—H	H···*A*	*D*···*A*	*D*—H···*A*
C2—H2···Cg3^i^	0.93	2.84	3.778 (4)	148
C14—H14···Cg2^ii^	0.93	2.97	3.658 (5)	147

Symmetry codes: (i) *x*+1/2, −*y*+1/2, *z*+1/2; (ii) −*x*+1, *y*, −*z*+1/2.
